# Effects of Pectin on the Physicochemical Properties and Freeze-Thaw Stability of Waxy Rice Starch

**DOI:** 10.3390/foods10102419

**Published:** 2021-10-13

**Authors:** Yuheng Zhai, Jiali Xing, Xiaohu Luo, Hao Zhang, Kai Yang, Xingfeng Shao, Kaihe Chen, Yanan Li

**Affiliations:** 1 Key Laboratory of Carbohydrate Chemistry and Biotechnology, Ministry of Education, Jiangnan University, Wuxi 214122, China; 6190112167@stu.jiangnan.edu.cn (Y.Z.); leslie198907@foxmail.com (H.Z.); yangkai164@outlook.com (K.Y.); lyn19881012ph@163.com (Y.L.); 2 National Engineering Laboratory for Cereal Fermentation Technology, Jiangnan University, Wuxi 214122, China; 3 Ningbo Academy of Product and Food Quality Inspection (Ningbo Fibre Inspection Institute), Ningbo 315048, China; hellojiali77@gmail.com; 4 College of Food and Pharmaceutical Sciences, Ningbo University, Ningbo 315211, China; shaoxingfeng@nbu.edu.cn; 5 Ningbo Gang Yagou Food Co., Ltd., Ningbo 315205, China; khchen0825@163.com

**Keywords:** waxy rice starch, pectin, physicochemical properties, freeze-thaw stability

## Abstract

In this study, the effects of the addition of pectin (PEC) on the physicochemical properties and freeze-thaw stability of waxy rice starch (WRS) were investigated. As PEC content increased, the pasting viscosity and pasting temperature of WRS significantly increased (*p* < 0.05), whereas its breakdown value and setback value decreased. Differential scanning calorimetry showed that the addition of PEC increased the gelatinization temperature of WRS, but decreased its gelatinization enthalpy. Rheological measurements indicated that the addition of PEC did not change the shear-thinning behavior of WRS–PEC blends, and the storage modulus and loss modulus were positively correlated with PEC content. Moreover, the textural parameter of WRS decreased with the increase in PEC content. Furthermore, the addition of PEC decreased the transmittance of starch paste, but enhanced the freeze-thaw stability of WRS to some extent. These results may contribute to the development of WRS-based food products.

## 1. Introduction

Waxy rice, also known as sticky or glutinous rice, is an important material for various food products, such as tangyuan (glutinous rice ball) and zongzi (glutinous rice dumplings) in Asian countries—especially China [1,2]. Waxy rice starch (WRS), which mainly contains amylopectin (over 98% of total starch), is considered to be an excellent food chemical raw material because of its viscosity, porous structure, and good water stability [3]. However, native WRS has the characteristics of insolubility in cold water, poor emulsification, easy aging, poor mechanical stability, and poor storage stability, limiting the application range of WRS [4]. According to previous studies, blends of starch with hydrocolloids could improve the properties of native starch and expand its functions [5,6,7,8].

Hydrocolloids such as carboxymethyl cellulose (CMC), guar, and xanthan gum have been widely used in starch-containing foods to modify their pasting and rheological properties and maintain the overall quality of the final product [9]. Chen et al. [10] claimed that pullulan can greatly affect the viscoelastic properties of rice starch gel systems, and that the expansion and gelatinization of rice starch could be inhibited when the amount added was greater than 0.07%. Nagano et al. [11] found that adding guar gum to corn starch could inhibit the leaching of starch components from starch granules during gelatinization. Liu et al. [12] found that the addition of kappa carrageenan could restrain the swelling of WRS granules during the gelatinization process, and finally form a gel with a homogenous network structure. The source of starch and the type of colloid used affect the physicochemical properties of starch to a great extent.

Pectin (PEC) is a kind of natural polysaccharide that exists widely in the primary wall and intermediate layer of plant cell walls. D-galacturonic acid is its basic binding unit, which is connected in a long chain with α-1,4 bonds [13]. In general, PEC can be divided into high-methoxyl PEC (50–80%) and low-methoxyl PEC (below 50%), according to its degree of methylation. As a water-soluble dietary fiber, PEC can enhance gastrointestinal peristalsis and promote nutrient absorption. PEC is also a typical hydrocolloid widely utilized in the food industry as a gelling, stabilizing, emulsifying, and thickening agent [14,15]. Therefore, many studies have applied PEC to influence the physicochemical properties of starch. It has been reported that PEC can enhance the stability of corn starch (CS) pasting, and CS–PEC mixtures exhibit a pseudoplastic and shear-thinning behavior [16]. Nawab et al. [17] also suggested that PEC could enhance the water-absorption ability of cowpea starch and reduce its swelling power and solubility; they added that PEC is a good improver for controlling syneresis during freeze-thaw cycles.

However, the existing scientific reports on the effects of PEC on starch properties are limited. Therefore, this study aimed to investigate the effects of different PEC concentrations on the pasting, thermal, rheological, and textural properties as well as freeze-thaw stability of WRS. This study may provide a theoretical basis for the potential utilization of hydrocolloids in improving the processing characteristics and storage stability of starch-based foods.

## 2. Materials and Methods

### 2.1. Materials

Waxy rice flour was obtained from Kemen Noodle Manufacturing Co., Ltd. (Yiyang, China). PEC extracted from apples was purchased from Youbaojia Food Co., Ltd. (shangqiu, China). The degree of esterification of PEC was 13.5%. Other analytical chemicals were purchased from China Pharmaceutical Chemicals Ltd. (Shanghai, China).

### 2.2. Isolation of Waxy Rice Starch

WRS was isolated in accordance with a previously published method, with some modifications [18]. Briefly, 10.0 g of waxy rice flour was dispersed in 100 mL of 0.4% NaOH solution for 48 h. The solution was changed every 12 h. The suspension was passed through a 200-mesh sieve and then centrifuged at 6000× *g* for 10 min. The upper gray matter (top layer) was scraped off, and the precipitate was washed five times with deionized water. pH was adjusted to 7.0 with 0.1 mol/L HCl. Afterward, the mixture was centrifuged at 6000× *g* for 20 min, and the precipitate was freeze-dried. The amylose, lipid, protein, and moisture contents of the WRS were 1.14%, 0.43%, 0.10%, and 7.57%, respectively.

### 2.3. Pasting Properties

The pasting properties of WRS with or without PEC were measured using a rapid visco-analyzer (RVA 4500, Perten Co. Ltd., Sydney, Australia) according to the method of Xie et al. [19], with some modifications. In brief, 3.0 g of WRS was added to an aluminum container, and different concentrations (0%, 0.1%, 0.2%, 0.3%, 0.4%, and 0.5%, *w*/*v*) of PEC solution were supplied to make up the final weight of 28 g. The suspension was equilibrated at 50 °C for 1 min and then heated to 95 °C within 3.5 min. The paste was held at 95 °C for 2.5 min. Subsequently, the hot sample was cooled to 50 °C within 4 min, and maintained at that temperature for 2 min. The paddle speed was set to 960 rpm for the first 10 s to disperse the sample, and then it was set to 160 rpm during the measurements.

### 2.4. Thermal Properties

The thermal properties of the sample were determined using a differential scanning calorimeter (DSC3, Mettler Toledo, Greifensee, Switzerland) in accordance with the method of Tao et al. [20], with some modifications. WRS samples (5 g, dry basis) were weighed and dispersed in 10 mL of distilled water or different concentrations (0.1%, 0.2%, 0.3%, 0.4%, and 0.5%) of PEC solution. The sample without PEC was used as a control. Afterward, the mixture was incubated using a vortex mixer (VORTEX 1, IKA (Guangzhou) Instrument Equipment Co., Ltd., Zhengzhou, China), quantitatively moved into a DSC pan, and sealed immediately. The samples were equilibrated at 4 °C for 12 h before testing, and the pans were heated from 30 °C to 100 °C at a rate of 10 °C/min.

### 2.5. Rheological Properties

#### 2.5.1. Frequency Sweep Test

**Figure 1:**
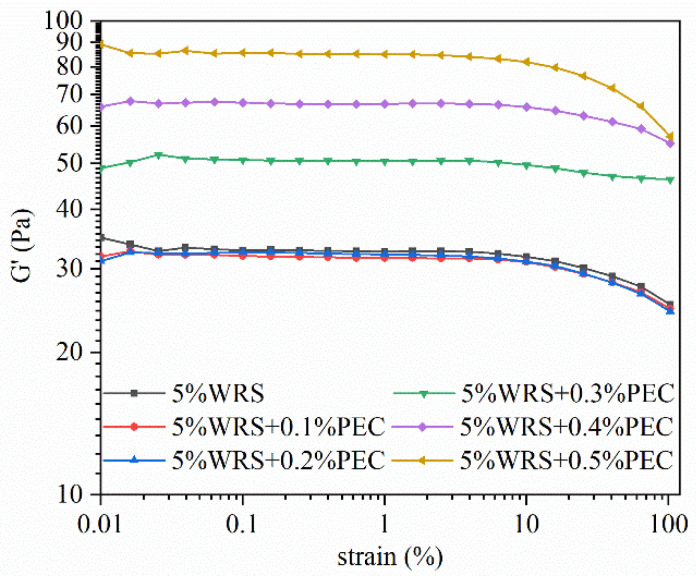
Strain sweep curves of the WRS and the WRS–PEC mixtures (frequency was set to 1 Hz).

The rheological properties of the gelatinized WRS–PEC mixtures obtained from RVA (rapid viscosity analyzer) were measured using a DHR-3 rheometer (TA Instruments Inc., New Castle, DE, USA). The samples were transferred to a rheometer plate and equilibrated at 25 °C for 1 min before each test. A 40 mm parallel plate with a gap of 1 mm was selected for the test. The frequency sweep test was performed at 25 °C within the range of 0.1–10 Hz, and the strain for measurements was selected as 1%, which was in the linear viscoelastic region according to the results of the strain sweep test (Figure 1).

#### 2.5.2. Flow Sweep Test

Steady shear viscosity was measured within the shear rate of 0.01–10 s^−1^ [21]. The following power-law model was used to fit the experimental curves:

(1) $$\tau =K\cdot \gamma^{n}$$

where τ is the shear stress (Pa), γ indicates the shear rate, K denotes the consistency index (Pa·sn), and n represents the flow behavior index.

### 2.6. Paste Clarity

The paste clarity of the WRS and the WRS–PEC mixtures was determined following the method of Lutfi et al. [22], with some modifications. The samples (2%, *w*/*v*) were heated in a boiling water bath for 30 min and then vortex-oscillated every 5 min. The samples were allowed to cool to room temperature (25 °C), and the clarity (% transmittance at 650 nm) of the samples was then measured using a UV-2100 spectrophotometer (UNICO Instrument Co., Ltd., Shanghai, China). Distilled water was set as a reference (100%).

### 2.7. Textural Properties

The textural properties of the WRS and the WRS–PEC mixtures were assessed using a TA.XTplus texture analyzer (Stable Micro Systems, Godalming, UK) according to a previous method, with some modifications [23]. The pastes obtained from RVA were immediately transferred into plastic cups with a cover (30 mm diameter, 20 mm height) and stored at 4 °C overnight. The samples were tested on the sample table of the texture analyzer, and the test conditions were set as follows: the P25 probe was selected, the pre-test and test speed was 1 mm/s, and the post-test speed was 5 mm/s. The compression depth was 10 mm per test, and the time between two compressions was 2 s.

### 2.8. Freeze-Thaw Stability

The freeze-thaw stability of the WRS and the WRS–PEC pastes was determined in accordance with the method of Tao et al. [20], with some modifications. WRS pastes with or without PEC were prepared as described in Section 2.6. The final concentration of both samples was 5% (*w*/*v*). The slurry was allowed to cool to room temperature, and then poured into a pre-weighed centrifuge tube to record its total weight. The samples were then frozen at −20 °C for 20 h, thawed at 25 °C for 3.5 h, and centrifuged at 5000× *g* for 15 min. The supernatant was poured out and weighed. These steps were repeated six times, and the mass of the centrifuge tube was recorded after each cycle. The rate of syneresis was calculated by the percentage of the supernatant weight to the gelatinized gel weight. The degree of syneresis (%) was calculated using Equation (2):

(2) $$\mathrm{syneresis}\;\left(\%\right)=\frac{\mathrm{Weight}\;\mathrm{of}\;\mathrm{released}\;\mathrm{liquid}}{\mathrm{Weight}\;\mathrm{of}\;\mathrm{starch}\;\mathrm{paste}}\times 100$$

### 2.9. Statistical Analysis

Data were expressed as mean ± standard deviation, and each test was carried out at least three times. Statistical significance was analyzed by one-way analysis of variance (ANOVA) using SPSS 16.0 (SPSS Inc., Chicago, IL, USA). The value of significance was set at *p* < 0.05.

## 3. Results and Discussion

### 3.1. Pasting Properties

The pasting properties of WRS with or without PEC, as measured by RVA, are presented in Table 1. The peak and final viscosity of WRS were 3415.5 and 2228.5 cP, respectively. The addition of PEC significantly (*p* < 0.05) changed the pasting profiles of the WRS–PEC mixtures. As PEC content increased from 0.1% to 0.5%, the peak and final viscosity of the WRS–PEC mixtures gradually increased from 3454.0 cP to 3548.5 cP and from 2244.0 cP to 2362.5 cP, respectively. This may be due to the possible interaction between starch molecules and hydrocolloids during the gelatinization process [10]. The interaction between PEC and amylopectin—especially the long exterior chains of amylopectin—may be the main reason for the increase in viscosity. However, the viscosity value of PEC (0.5%, m/v) solution was within the range of 13–20 cP (Figure S1), which was substantially lower than that of WRS and the WRS–PEC mixtures. Thus, the viscosity of PEC itself had a negligible effect on overall viscosity. In general, the breakdown represents the degree of disintegration of starch granules, and a better paste stability normally leads to a lower breakdown value [14]. The breakdown value of pure WRS was 1761.0 cp. However, the addition of PEC gradually decreased the breakdown value of the mixtures, i.e., breakdown decreased from 1633.0 cP to 1551.0 cP when PEC content increased from 0.1% to 0.5%. The decrease in breakdown values might due to the PEC being able to cover the starch granules step by step with the increasing concentration of PEC, so that the stability of the mixtures was enhanced. By comparison, the setback value reflects the difference in viscosity between the hot paste and the gel formed after cooling, and it is an indicator of starch retrogradation [24]. The setback value of pure WRS was 574.0 cp. The addition of PEC significantly (*p* < 0.05) reduced the setback of the WRS–PEC mixtures, implying that PEC inhibited the retrogradation of gelatinized starch. Moreover, the pasting temperature of the WRS–PEC mixtures was enhanced by the addition of PEC, but not significantly (*p* > 0.05). This observation might be attributed to the competition between PEC and starch granules for water absorption, which inhibited the expansion of starch granules to improve the pasting temperature [22].

### 3.2. Thermal Properties

**Figure 2:**
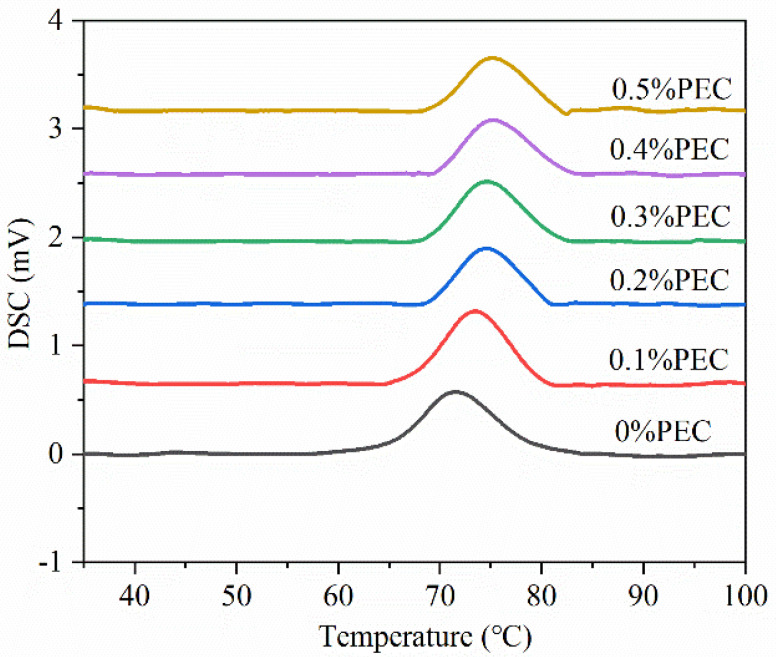
Thermograms of the WRS and the WRS–PEC mixtures.

The DSC thermograms of WRS with different PEC contents are presented in Figure 2, and the derived thermal parameters are summarized in Table 2. As expected, a single thermogram was observed for pure WRS. The estimated onset temperature (*T_o_*), peak temperature (*T_p_*), conclusion temperature (*T_c_*), and enthalpy (Δ*H*) were 65.38 °C, 71.63 °C, 78.50 °C, and 6.20 J/g, respectively. After the addition of PEC, the thermograms gradually shifted to higher temperatures as PEC content increased from 0.1%, 0.2%, 0.3%, and 0.4% to 0.5%, corresponding to the *T_p_* at 72.53 °C, 73.92 °C, 74.22 °C, 74.55 °C, and 74.81 °C, respectively. Furthermore, the addition of PEC gradually increased the *T_o_* of the blends, which was consistent with the results of the RVA analysis. However, the *T_o_* of the blends was lower than the pasting temperature measured using the RVA, in accordance with the findings of Shahzad et al. [25], who speculated that the melting process could result in an early increase in viscosity, and might be attributed to the properties measured by two different methods. Overall, these observations indicate that the addition of PEC improved the thermal stability of WRS, in accordance with the results reported for barley starch/PEC [22] and potato starch/PEC systems [26]. In general, a lower extent of starch hydration results in a higher gelatinization temperature. Thus, the addition of PEC might reasonably compete with starch granules for water absorption, thereby decreasing the hydration of starch granules and enhancing the thermal stability of WRS. As another possibility, the structure of PEC contains numerous hydroxyl groups that interact with starch to form stable hydrogen bonds, resulting in thermally stable WRS–PEC blends. The Δ*H* of the WRS–PEC blends decreased from 5.34 J/g to 3.09 J/g as the PEC content increased from 0.1% to 0.5% (Table 2). According to Liu et al. [27], the Δ*H* for starch gelatinization primarily reflects the energy consumed for helix dissociation and uncoiling. The decrease in Δ*H* may be due to the high hydrophilicity of PEC molecules which, in turn, could decrease water diffusion within WRS granules and lead to incomplete starch gelatinization [8]. Moreover, the interactions between PEC and WRS molecules might change the coupling force between microcrystalline and amorphous matrices, thereby further reducing the required energy for melting [28].

### 3.3. Dynamic Rheological Properties

**Figure 3:**
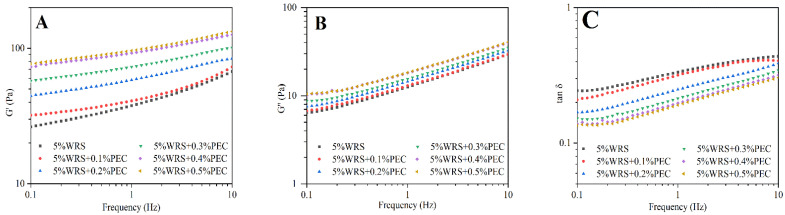
Variation of *G*’ (**A**), *G*” (**B**), and tan δ (**C**) with frequency for the WRS and the WRS–PEC mixtures.

Dynamic modulus can be used to examine the interactions between the dispersed phase and the continuous phase in some polymer solutions [21]. In general, *G*’ represents the energy stored as mechanical energy, whereas *G*” measures the energy dissipated as heat, and mostly represents the viscous portion [29]. As shown in Figure 3A,B, the *G*’ and *G*” of all samples increased as the scanning frequency increased from 0.1 Hz to 10 Hz. Moreover, the value of *G*’ was slightly higher than that of *G*”, indicating that both WRS and the WRS–PEC blends had weak gel properties. As the PEC content increased, the *G*’ and *G*” of the blends correspondingly increased. The phase separation caused by the incompatibility between PEC and WRS in the continuous phase likely increased the effective starch concentration by fixing water molecules, and a high PEC content could lead to a more rigid gel [30]. The tan δ value is calculated as the viscous modulus divided by the elastic modulus, and reflects the comprehensive viscoelastic properties of WRS and WRS–PEC blends. The tan δ of all of the samples was less than 1, whereas the tan δ of the blends decreased as the PEC content increased (Figure 3C), indicating that PEC transformed the gel network of WRS from viscous to solid. Similar phenomena have been observed in mixtures of PEC and other starches [16,23].

### 3.4. Steady Shear Rheological Properties

**Figure 4:**
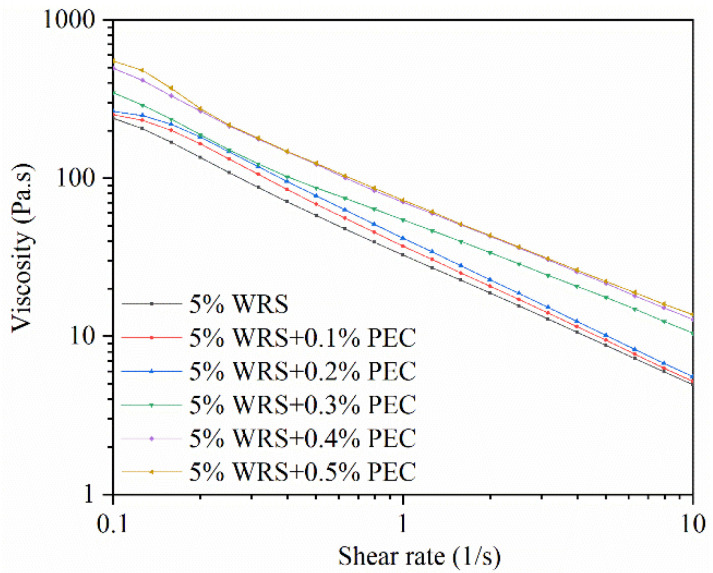
Steady shear of the WRS and the WRS–PEC mixtures.

The steady flow curves of the WRS–PEC blends are shown in Figure 4. As the shear rate increased, the apparent viscosity of all of the samples gradually decreased, indicating that the starch pastes with and without PEC all exhibited non-Newtonian shear-thinning flow behavior. Similar results have been reported for other starch–hydrocolloid systems [10,21]. Moreover, the addition of PEC significantly changed the steady rheological properties of the WRS–PEC blends (*p* < 0.05). Furthermore, a higher level of PEC resulted in a higher value of apparent viscosity. As PEC content increased, hydrogen bonds were formed between PEC and WRS. At the same time, the hydroxyl groups of PEC competed with WRS for water molecules [20], which reduced water activity and inhibited the movement of starch chains, thereby increasing the viscosity of the WRS–PEC blends.

The power-law model was applied to describe the flow properties of the blends. The fitted parameters are listed in Table 3. All of the determination coefficients (R^2^) were within the range of 0.9759–0.9991, indicating that the power-law model fitted the rheological behavior of the samples well. The flow behavior index (n) reflects the closeness to a Newtonian fluid: n = 1 corresponds to a Newtonian fluid, whereas n < 1 represents a higher degree of pseudoplasticity [31]. The n value of WRS alone was 0.11, and the addition of PEC changed the flow behavior of the WRS–PEC blends, i.e., the n value increased from 0.13 to 0.24 as PEC content increased from 0.1% to 0.5%. All of the n values were less than 1, suggesting that all of the pastes exhibited pseudoplastic behavior. The consistency coefficient (K) represents changes in apparent viscosity. The higher the K value, the higher the viscosity. The K value increased from 41.36 Pa·s^n^ to 74.86 Pa·s^n^ as the PEC content increased (Table 3). The results indicate that the thickening of the mixed system was enhanced, and that the pseudoplasticity of the starch–hydrocolloid blends decreased as the PEC content increased. Similar findings were reported for pearl millet starch/guar gum gels [8].

### 3.5. Paste Clarity

Transparency is an indicator that describes the behavior of starch paste in terms of light transmission; it is influenced by particle size, amylose content, amylose/amylopectin ratio, swelling capability, and residual degree of swollen and non-swollen particles [20]. The transparency of WRS with or without PEC pastes is shown in Table 3. The transparency of WRS paste was 7.0%. As PEC content increased from 0.1% to 0.5%, the transmittance value significantly decreased from 6.17% to 1.37% (*p* < 0.05). Yousefi et al. [32] reported similar results for starch–gum blends—they found that the paste clarity of wheat starch decreased as the content of sage seed gum increased. Transparency increases with the expansion of starch granules [33]. As PEC content increased, PEC was further wrapped around the starch particles to compete with starch for water absorption, thus inhibiting the expansion of the WRS granules and reducing the transparency of the pastes.

### 3.6. Textural Properties

The textural properties of starch gels are the sensory indicators for evaluating the chewing of starch-based foods, so these characteristics are important criteria for determining the properties of starch in food systems [34]. It can be seen from Table 4 that the hardness, adhesiveness, springiness, cohesiveness, and gumminess of pure WRS gel were 206.26 g, 66.51 g·s, 1.28, 0.92, and 114.94 g, respectively. It could be observed that the hardness of WRS–PEC gels was significantly decreased from 197.34 g to 137.63 g (*p* < 0.05) with the increase in PEC. Hardness is mainly caused by the retrogradation of starch gel, and is affected by amylose and amylopectin chain rearrangement [35]. Therefore, the decrease in hardness with increasing PEC concentration could be due to PEC forming hydrogen bonds with WRS molecules, which interfered with the formation of ordered structures during starch retrogradation. Similarly, adhesiveness and gumminess also significantly decreased from 60.05 g·s to 32.78 g·s and 103.98 g to 75.06 g, respectively (*p* < 0.05), with the increase in the added PEC ratio. Adhesiveness is related to the force required to move substances attached to the mouth, and gumminess directly represents the energy required for food decomposition before swallowing [34]. The decrease in adhesiveness and gumminess with the increase in PEC content might be attributed to the possibility that PEC could cover the starch molecules, resulting in reduced starch–starch hydrophobic interaction. Lower hydrophobic interaction leads to inhomogeneity and instability in the network structure of the gels, reducing the textural characteristics [36].

However, there were no significant differences (*p* < 0.05) in springiness and cohesiveness after adding PEC to the WRS. The springiness of the gel represents its ability to recover from distortion [25]. After adding PEC, a slight reduction was found for springiness, which means that the gels were easy to chew.

### 3.7. Freeze-Thaw Stability

**Figure 5:**
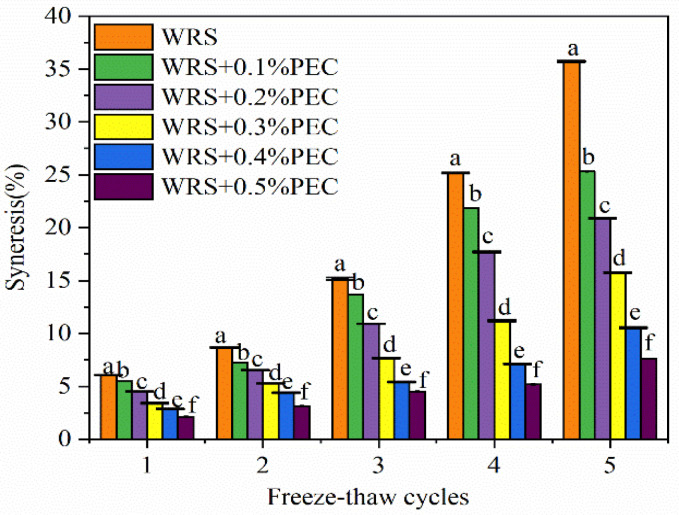
Effect of PEC on the freeze-thaw stability of the WRS and the WRS–PEC mixtures. Means with different small letter superscripts are significantly different at *p* < 0.05.

The freeze-thaw stability is an important factor reflecting the stability of food quality; it is used to evaluate the ability of starch to resist adverse physical changes caused by freeze-thaw cycles [37,38]. When the water in starch gel forms ice crystals during freezing, the phase separation occurs in the system. After thawing, water is easily expelled from the dense gel network, causing the starch paste to dehydrate [37]. The syneresis of WRS with or without PEC pastes during five freeze-thaw cycles is shown in Figure 5. After five freeze-thaw cycles, the syneresis of WRS increased from 6.08% to 35.73%. The syneresis of the WRS–PEC blends with 0.1%, 0.2%, 0.3%, 0.4%, and 0.5% PEC content significantly increased from 5.52% to 25.31%, from 4.52% to 20.87%, from 3.41% to 15.76%, from 2.85% to 10.54%, and from 2.11% to 7.63%, respectively (*p* < 0.05). Repeated freeze-thaw cycles promote the growth and phase separation of ice crystals, and accelerate the binding of intermolecular hydrogen bonds, resulting in sponge-like structures and the release of water [20].

Although the freeze-thaw cycles increased the syneresis of the WRS–PEC blends, the addition of PEC seemed to inhibit the increase in the syneresis of these blends. For example, after five freeze-thaw cycles, the syneresis of the WRS–PEC blends decreased from 35.73% to 7.63% as PEC content increased from 0.1% to 0.5%. This difference might be due to the high water-holding capacity of PEC, which could prevent the release of water molecules from the matrix and control the growth of ice crystals in the system. Overall, the results indicate that PEC can inhibit the dehydration of starch gels during freeze-thaw cycles, thereby improving the quality of starch-based food products.

## 4. Conclusions

The effects of PEC on the pasting, thermal, rheological, and textural properties, as well as on the freeze-thaw stability of the WRS–PEC mixtures, were investigated. The RVA results showed that as the PEC concentration increased, the peak viscosity, final viscosity, and pasting temperature of the WRS–PEC mixtures increased, whereas their breakdown and setback values decreased. DSC results showed that the addition of PEC substantially increased the gelatinization temperature and decreased the gelatinization enthalpy of starch. Rheological tests revealed that the WRS and the WRS–PEC mixtures were pseudoplastic fluids, and that their moduli (*G*’ and *G*”) increased with the increase in PEC concentration. The textural parameters of WRS decreased with the increase in the concentration of PEC. Analyses of paste clarity showed that the addition of PEC promoted the swelling of WRS to some extent. Moreover, the freeze-thaw stability of WRS was enhanced by the addition of PEC. In conclusion, this study provides theoretical support for the improvement of WRS quality by adding PEC, and suggests possible applications of PEC in WRS-based products.

## Figures and Tables

**Table 1:** Pasting properties of the WRS and the WRS–PEC mixtures *.

Pectin Concentration %	Peak Viscosity (cP)	Trough (cP)	Final Viscosity (cP)	Breakdown (cP)	Setback (cP)	Pasting Temperature (°C)
0	3415.5 ± 0.5 ^f^	1654.5 ± 1.5 ^f^	2228.5 ± 2.5 ^f^	1761.0 ± 1.0 ^a^	574.0 ± 1.0 ^a^	75.2 ± 0.4 ^d^
0.1	3454.0 ± 1.0 ^e^	1821.0 ± 2.0 ^e^	2244.0 ± 3.0 ^e^	1633.0 ± 1.0 ^b^	423.0 ± 1.0 ^b^	76.7 ± 0.4 ^c^
0.2	3478.5 ± 1.5 ^d^	1884.0 ± 3.0 ^d^	2294.5 ± 2.5 ^d^	1594.5 ± 1.5 ^c^	410.5 ± 0.5 ^c^	77.1 ± 0.0 ^b, c^
0.3	3505.0 ± 2.0 ^c^	1927.5 ± 2.5 ^c^	2320.0 ± 3.0 ^c^	1577.5 ± 0.5 ^d^	392.5 ± 0.5 ^d^	78.0 ± 0.1 ^a, b^
0.4	3529.0 ± 2.0 ^b^	1966.5 ± 3.5 ^b^	2348.0 ± 3.0 ^a^	1562.5 ± 1.5 ^e^	381.5 ± 0.5 ^e^	78.2 ± 0.3 ^a^
0.5	3548.5 ± 1.5 ^a^	1997.5 ± 3.5 ^a^	2362.5 ± 2.5 ^a^	1551.0 ± 5.0 ^f^	365.0 ± 1.0 ^f^	78.7 ± 0.0 ^a^

* Values are means ± SD. Identical letters in the same column represent significant differences (*p* < 0.05).

**Table 2:** Thermal parameters of the WRS and the WRS–PEC mixtures *.

Pectin Concentration (%)	*T_o_* (°C)	*T_p_* (°C)	*T_c_* (°C)	Δ*H* (J/g)
0	65.38 ± 0.34 ^f^	71.63 ± 0.31 ^e^	78.50 ± 0.15 ^c^	6.20 ± 0.02 ^a^
0.1	66.84 ± 0.12 ^e^	72.53 ± 0.03 ^d^	77.26 ± 0.03 ^b^	5.34 ± 0.01 ^b^
0.2	68.01 ± 0.12 ^d^	73.92 ± 0.01 ^c^	78.65 ± 0.01 ^b^	4.68 ± 0.02 ^c^
0.3	69.77 ± 0.16 ^c^	74.22 ± 0.16 ^b, c^	79.28 ± 0.14 ^a, b^	4.26 ± 0.01 ^d^
0.4	68.92 ± 0.11 ^b^	74.55 ± 0.11 ^a, b^	79.56 ± 0.28 ^a^	3.24 ± 0.01 ^e^
0.5	70.90 ± 0.06 ^a^	74.81 ± 0.10 ^a^	79.72 ± 0.45 ^a^	3.09 ± 0.01 ^f^

* Values are means ± SD. Identical letters in the same column represent significant differences (*p* < 0.05).

**Table 3:** Power-law parameters and transmittance of WRS pastes with different pectin contents *.

PEC Concentration (%)	K (Pa.s^n^)	n	R^2^	Transmittance (%)
0	41.36 ± 2.07 ^d^	0.11 ± 0.01 ^b^	0.976	7.00 ± 0.33 ^a^
0.1	42.7 4± 0.98 ^c, d^	0.13 ± 0.02 ^b^	0.982	6.17 ± 0.42 ^b^
0.2	46.74 ± 1.20 ^c^	0.14 ± 0.01 ^b^	0.985	4.63 ± 0.19 ^c^
0.3	57.54 ± 0.92 ^b^	0.25 ± 0.00 ^a^	0.988	3.93 ± 0.09 ^d^
0.4	72.12 ± 0.71 ^a^	0.26 ± 0.01 ^a^	0.999	2.43 ± 0.21 ^e^
0.5	74.86 ± 0.48 ^a^	0.24 ± 0.00 ^a^	0.997	1.37 ± 0.12 ^f^

* Values are means ± SD. Identical letters in the same column represent significant differences (*p* < 0.05).

**Table 4:** Textural parameters of the WRS pastes with different pectin contents *.

PEC Concentration	Hardness	Adhesiveness	Springiness	Cohesiveness	Gumminess
(%)	(g)	(g·s)			(g)
0	206.26 ± 5.84 ^a^	66.51 ± 1.32 ^a^	1.28 ± 0.00 ^a^	0.94 ± 0.03 ^a^	114.94 ± 1.95 ^a^
0.1	197.34 ± 1.37 ^b^	60.05 ± 3.84 ^b^	1.27 ± 0.01^a, b^	0.93 ± 0.01 ^a^	103.98 ± 2.41 ^b^
0.2	187.51 ± 1.54 ^c^	52.66 ± 2.87 ^c^	1.26 ± 0.00 ^a, b^	0.92 ± 0.01 ^a^	101.72 ± 1.78 ^b^
0.3	186.58 ± 2.76 ^c^	44.95 ± 1.74 ^d^	1.24 ± 0.01 ^a, b, c^	0.88 ± 0.01 ^b^	98.84 ± 2.77 ^b^
0.4	157.00 ± 2.14 ^d^	36.62 ± 1.46 ^e^	1.25 ± 0.01 ^b, c^	0.86 ± 0.02 ^b^	90.63 ± 3.09 ^c^
0.5	137.63 ± 2.19 ^e^	32.78 ± 0.74 ^e^	1.21 ± 0.04 ^c^	0.85 ± 0.01 ^b^	75.06 ± 3.51 ^d^

* Values are means ± SD. Identical letters in the same column represent significant differences (*p* < 0.05).

## Data Availability

Data available on request due to privacy.

## Supplementary Materials

The following are available online at https://www.mdpi.com/article/10.3390/foods10102419/s1, Figure S1: Rapid Visco-Analyzer (RVA) pasting profiles of PEC solution (0.5%, *w*/*v*).
