# Supplementary figures and images for: Effects of Pectin on the Physicochemical Properties and Freeze-Thaw Stability of Waxy Rice Starch
Source: Foods. 2021 Oct 13;10(10):2419. doi: 10.3390/foods10102419 (PMC8536014; doi:10.3390/foods10102419)

## Supplementary material

**Figure S1**

Rapid Visco-Analyzer (RVA) pasting profiles of PEC solution (0.5%, w/v).

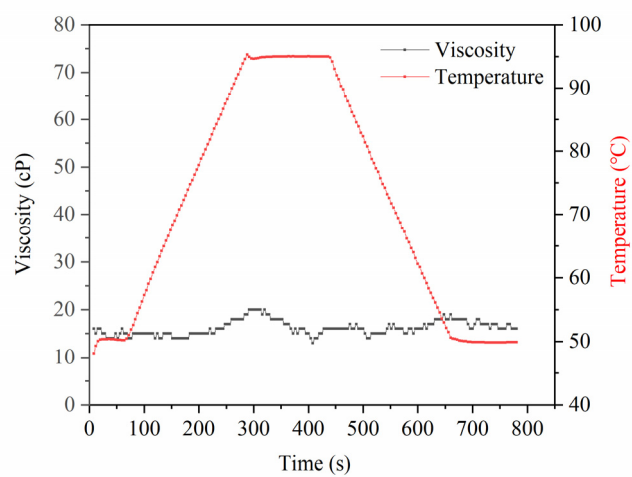

Supplement: Supplementary file 1 [file foods-10-02419-s001.zip › foods-1374596-supplementary.pdf]
